# Fatal acute undifferentiated febrile illness among clinically suspected leptospirosis cases in Colombia, 2016–2019

**DOI:** 10.1371/journal.pntd.0011683

**Published:** 2023-10-16

**Authors:** Eliana L. Parra Barrera, Jhonatan Reales-González, Daniela Salas, Elizabeth Reyes Santamaría, Solmara Bello, Angélica Rico, Lissethe Pardo, Edgar Parra, Karina Rodriguez, Zonía Alarcon, Angela Patricia Guerra Vega, Mayra A. Porras, Sergio Yebrail Gomez-Rangel, Carolina Duarte, Jaime Moreno

**Affiliations:** 1 Grupo de Microbiología, Subdirección Laboratorio Nacional de Referencia. Dirección de Redes en Salud Pública, Instituto Nacional de Salud, Bogotá, Colombia; 2 Grupo de Virología, Subdirección Laboratorio Nacional de Referencia. Dirección de Redes en Salud Pública, Instituto Nacional de Salud, Bogotá, Colombia; 3 Grupo de Microbiología, Subdirección de Investigación en Salud Pública. Instituto Nacional de Salud, Bogotá, Colombia; 4 Grupo de Enfermedades Transmitidas por Vectores y Zoonosis, Instituto Nacional de Salud, Bogotá, Colombia; 5 Departamento de Medicina interna y Departamento de Medicina crítica y cuidados intensivos. Hospital Universitario Fundación Santa Fe de Bogotá, Colombia; 6 Grupo de Enfermedades Transmisibles Prevenibles por Vacunación en Salud, Dirección de Vigilancia y Análisis del Riesgo en Salud Pública, Instituto Nacional de Salud, Bogotá, Colombia; 7 Grupo de Patología, Subdirección Laboratorio Nacional de Referencia. Dirección de Redes en Salud Pública, Instituto Nacional de Salud, Bogotá, Colombia; 8 Grupo de Parasitología, Subdirección Laboratorio Nacional de Referencia. Dirección de Redes en Salud Pública, Instituto Nacional de Salud, Bogotá, Colombia; Tufts Medical Center, UNITED STATES

## Abstract

**Background:**

Acute undifferentiated febrile illness is a common challenge for clinicians, especially in tropical and subtropical countries. Incorrect or delayed diagnosis of febrile patients may result in medical complications or preventable deaths. Common causes of acute undifferentiated febrile illness in Colombia include leptospirosis, rickettsioses, dengue fever, malaria, chikungunya, and Zika virus infection. In this study, we described the acute undifferentiated febrile illness in postmortem patients reported as suspected cases of leptospirosis through the national leptospirosis surveillance in Colombia, 2016–2019.

**Methodology/principal findings:**

We retrospectively analyze human fresh and formalin-fixed tissue samples from fatal suspected leptospirosis cases reported by the Public Health Laboratories in Colombia. Leptospirosis confirmation was made by immunohistochemistry, real-time polymerase chain reaction (PCR) in the tissue samples. In some cases, the serum sample was used for confirmation by Microagglutination test (MAT). Simultaneously, tissue samples were tested by PCR for the most common viral (dengue, Zika, and chikungunya), bacterial (*Brucella* spp., and *Rickettsia* spp.), and parasitic (malaria). Fresh tissue samples from 92 fatal suspected leptospirosis cases were reported to the National Reference Laboratory from 22/32 departments in Colombia. We confirmed leptospirosis in 27% (25/92) of cases. Other pathogens identified by real-time PCR were *Brucella* spp. (10.9%), *Rickettsia* spp. (14.1%), and dengue (2.2%). Dengue (6.9%), hepatitis (3.5%), and Yellow Fever cases (2.2%) were detected by the pathology. All patients were negative for chikungunya and *Plasmodium* spp. Most cases were classified as undifferentiated febrile illnesses (45.7%; 42/92).

**Conclusions/Significance:**

This study underscores the importance of early and accurate recognition of leptospirosis to prevent mortalities. Moreover, it draws attention to the existence of other febrile syndromes in Colombia, including rickettsiosis and brucellosis, that currently lack sufficient human surveillance and regular reporting. Expanding laboratory surveillance to include viruses such as Hantavirus, Mayaro virus, Oropouche virus, and West Nile virus is crucial.

## Author summary

Leptospirosis is a potentially fatal disease caused by the bacteria *Leptospira*. Its signs and symptoms typically appear one to two weeks following infection, range from mild (e.g., fever, headache) to severe (e.g., meningitis). Despite knowledge of risk factors for leptospirosis, there are still significant gaps in knowledge about symptoms, infection routes, and risk. A correct and timely diagnosis of leptospirosis could potentially avoid medical complications or death but is challenging due to non-specific presentation and limited availability of diagnostics. This is particularly difficult in a tropical country where acute undifferentiated febrile illnesses can be caused by many pathogens. One such country is Colombia, where leptospirosis is endemic. In this study, we retrospectively study acute undifferentiated febrile illness in 92 *postmortem* cases reported as suspected leptospirosis cases through the national leptospirosis surveillance in Colombia, 2016–2019. Only a small proportion (27%) of cases were confirmed as leptospirosis; other etiologies including dengue, hepatitis, and yellow fever were found. However, 45.7% of cases were acute undifferentiated febrile illness, underscoring diagnostic limitations common in the tropics, and the need to recognize local pathogens and clinical characteristics to perform early presumptive diagnosis in cases with acute undifferentiated febrile illness.

## Introduction

Febrile illness is among the most common reasons for people seeking healthcare globally [1–3]. Acute undifferentiated febrile illness (AUFI) can be caused by a wide range of causes, including several emergent and re-emergent pathogens of global importance, causing substantial morbidity and mortality in low- and middle-income countries [1,2,4–6]. The proportion of AUFI in low-and middle-income countries is high, and most of these countries are endemic for a wide range of etiologies such as leptospirosis, rickettsioses, dengue fever, malaria, chikungunya, and Zika virus infection [2,4,6–10]. Many AUFI etiologies are preventable. However, their non-specific presentation, often mimicking each other, and the limited availability of diagnostic tools and laboratory services make accurate and timely diagnosis challenging [1,2]. Delayed diagnosis or inappropriate medical management may lead to medical complications or death [7,11].

Leptospirosis is an acute septicemic febrile disease caused by pathogenic species of the *Leptospira* genus, which affects humans and animals [12,13]. Globally, leptospirosis causes approximately 1.03 million cases annually (95% confidence intervals 0.43–1.75 million), with an estimated 58,900 deaths (95% CI: 23,800–95,900) [14]. The highest mortality is presented in poor-resource and tropical regions where the burden of leptospirosis has been under-appreciated compared to other febrile illnesses [15]. However, transmission occurs in high-income countries in temperate regions as well [12,16,17]. In humans, leptospirosis signs and symptoms range from subclinical infection to severe life-threatening manifestations [12,13]. The disease begins with a septicemic phase and immune manifestations, followed by a severe presentation with vascular, hepatic, renal, pulmonary, and skeletal muscle injury or Weil´s syndrome [12]. Weil´s syndrome, the most severe form of leptospirosis, is estimated for 5% to 15% of clinical leptospirosis cases [12,18]. Leptospirosis mortality probably depends on the host (e.g., age and sex) and bacterial factors (e.g., serovar), and varies upon clinical presentation, from 0% in patients with non-severe disease to over 50% for patients with chronic leptospirosis [19].

As a tropical country, Colombia presents many favorable risk factors for the transmission and spread of pathogens that cause AUFI [20–24]. Their non-specific clinical presentation and limited diagnostic tools and laboratory capabilities make it challenging to determine accurate diagnoses of AUFI. This leads to under-ascertainment and misclassification of cases, limited understanding of the incidence of etiologies, and fatal cases with an unclear diagnosis. Subclinical or asymptomatic infections, which may be linked to chronic disease, are also common in endemic areas [25,26]. Consequently, the burden of AUFI has not been thoroughly described in Colombia [20]. For decades, the country has presented a high proportion of reported dengue cases [22,27,28] and Malaria [29]. However, some suspected dengue cases have been misclassified and caused by other tropical etiologies such as leptospirosis, rickettsioses, chikungunya virus infection, and Zika virus infection [23,30].

Leptospirosis became a mandatory reportable disease in 2007 in Colombia [31]. Leptospirosis diagnostic screening is mainly based on serological tests or pathology diagnostic [31]. In 2015, healthcare institutions were encouraged to send fresh human tissues from clinically suspected leptospirosis deaths for diagnostic confirmation by real-time PCR in the Microbiology group of the National Institute of Health (INS in Spanish). This study aimed to describe the acute undifferentiated febrile illness in postmortem patients reported as suspected cases of leptospirosis through the national leptospirosis surveillance in Colombia, 2016–2019.

## Materials and methods

### Ethics statements

This study was part of the national public health surveillance program of the National Institute of Health (INS), a governmental agency reporting to the Colombian Ministry of Health. The surveillance program included an epidemiological form with information on demographic and leptospirosis risk factors. This study was reviewed and approved by Scientific Ethics Committee at the INS (Protocol CTIN 8–201, act number 1, 2016). Personal information (name, address, telephone, among other data that could be personally identifiable) was not used in the study. Informed consent was not required.

### Study and sample collection

**Fig 1:**
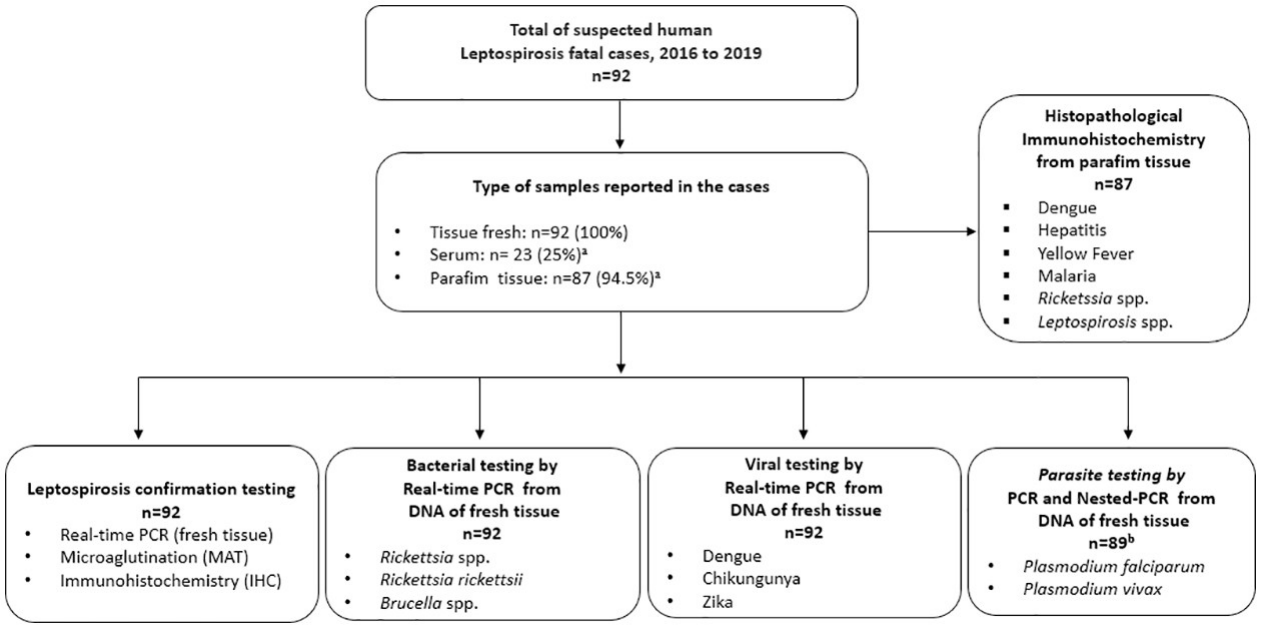
Study flow diagram with the distribution of suspected leptospirosis cases and other etiologies confirmation. ^a^Out of the selected suspected leptospirosis death cases, 92 had fresh tissue samples. However, paraffin tissue samples were reported in 87 cases and only 23 cases had available serum samples. ^b^The Malaria identification was possible in 89 fresh tissue samples, unfortunately, the available fresh tissue samples were not sufficient for DNA extraction.

We conducted a retrospective study of the analysis of human tissue samples from fatal clinically suspected leptospirosis. The fresh and paraffin tissue samples were sent through the Leptospirosis Laboratory Surveillance at INS, Colombia, from 2016 to 2019 (Fig 1).

Leptospirosis is a notifiable disease since 2007 in Colombia [31]; cases are reported to the national surveillance system SIVIGILA (Sistema Nacional de Vigilancia en Salud Pública). Surveillance involves three entities: i) healthcare institutions, including hospitals, medical clinics, and laboratories, ii) the Departmental Public Health Laboratory Network, which includes public health laboratories in each of the 32 departments of Colombia and oversees technical and administrative actions, public health surveillance and disease control, and quality management, and iii) the National Reference Laboratory, which coordinates the departmental laboratory network, and is responsible for confirming leptospirosis cases. Diagnostic screening is done from serum samples by a commercial Immunoglobulin M Enzyme-Linked Immunosorbent Assays, and cases are confirmed with Microscopic Agglutination Tests (MAT) and a pathology diagnosis [31].

Although leptospirosis diagnosis is based on MAT or pathology diagnostic, in 2015, the INS offered confirmation by real-time PCR. Departmental laboratories had to send fresh human tissues from fatal clinically suspected leptospirosis patients to the Microbiology group at INS. All samples were sent in separate vials and emulsified in a sterile buffered saline solution. Transportation was made using ice refrigerants, and samples were stored at -70°C until the diagnostic procedures started. Fresh human tissue samples included samples from the kidney, liver, lung, brain, cerebellum, heart, and spleen.

### Surveillance leptospirosis definitions

All samples were obtained from suspected leptospirosis cases. The leptospirosis case definition was based on patients’ symptomatology and epidemiological data, as established by the leptospirosis surveillance protocol for Colombia [32]. Leptospirosis is suspected in patients with fever (>38° C) during the three previous weeks, accompanied by two or more symptoms, including headache, myalgia, jaundice, arthralgia, conjunctivitis, vomiting, diarrhea, back pain, reticular pain, and rash. Epidemiologic factors are also considered, including exposure to potentially contaminated standing water and mud, contact with sick animals and rodents, high seasonal rainfall, and occupations considered hazardous, including garbage collection, streams or street cleaning, livestock farming and butchering, and agriculture (e.g., rice farming).

### Serological testing by Microscopic Agglutination Test (MAT)

Serum samples were analyzed using the MAT test to confirm the diagnosis of leptospirosis. The MAT employed in this study included six pathogenic species (*L*. *interrogans*, *L*. *borgpetersenii*, *L*. *weilii*, *L*. *kirschneri*, *L*. *noguchii*, *and L*. *santarosai*) and one non-pathogenic species (*L*. *biflexa*) as a control. These species represented a total of 20 serogroups, as detailed in S1 Table. Confirmed cases of acute leptospirosis were based on MAT criteria with titers of 1:400 in fatal cases or a fourfold or greater rise in MAT titers between acute-phase and convalescent-phase samples (taken at the onset of symptoms and 10 to 15 days after the initial serum sample) also indicates a reactive sample.

### Nucleic acid isolation

Specimens were processed under aseptic conditions to minimize contamination. Tissues were carefully sectioned, following stringent protocols to ensure sample integrity. DNA isolation was performed using approximately 1 gram of each tissue sample. Initially, the samples were lysed using 1.5 ml of lysis buffer, and 20 μl of proteinase K (Invitrogen) was added, followed by overnight incubation at 56 °C. Subsequently, 500 μl of the lysate was subjected to automated purification using the MagNA Pure 96 Instrument (Roche Molecular Systems, Inc.). The final elution step was performed with 100 μl of elution buffer, and the samples were stored at -20°C. To create a composite sample per case, the eluted samples from each tissue were mixed. Individual tissue samples were not tested separately.

### Real-time PCR and etiology detection

Various real-time PCR assays were conducted to detect the most common etiological agents in the tissue samples. Specifically, leptospirosis-related fatalities were confirmed using a real-time PCR assay developed by Galloway and Hoffmaster from the US Centers for Disease Control and Prevention (CDC). This established PCR method has been previously published and widely recognized for its accuracy and specificity in identifying leptospirosis cases [33]. The *lipL32* gene was targeted for amplification to detect pathogenic *Leptospira* spp. To ensure the accuracy of the amplification process, a commercial internal control (TaqMan Exogenous Internal Positive Control Reagents, Applied Biosystems) was incorporated into the PCR setup to verify the successful amplification of the sample. For the detection of *Rickettsia spp*., and *Rickettsia rickettsii* was used the real-time PCRs assays originally proposed by Kato et al [34], amplified the target 23S rRNA and a gene encoding hypothetical protein A1G_04230, respectively. To detect *Brucella* spp. infections, a real-time PCR to amplify the insertion sequence *IS711*, universally present in the different species of this genus, was used [35]. In both, the PCR to identify *Rickettsia* spp., and *Brucella* spp., were simultaneously carried out with an internal control, using a specific primer for the human ribonuclease gene to verify the DNA sample’s presence after extraction. Infections due to dengue, chikungunya, and Zika virus were detected and differentiated by using a triplex real-time RT-PCR (CDC) [36]. The detection of *Plasmodium* spp. was determined by PCR using the methodology reported by Pinheirob *et al*. [37,38]. A nested PCR was performed to the detection of *Plasmodium falciparum* and *Plasmodium vivax* [39]. To ensure the accuracy and reliability of the results, all the assays conducted in this study included a no template control (NTC). More descriptive information about the PCR is presented in the S2 Table.

### Histopathological study

Samples of human formalin-fixed tissues were subjected to postmortem histology analyses at the Pathology Laboratory, INS (Instituto Nacional de Salud). As per the leptospirosis surveillance protocol, tissues from fatal cases suspected of leptospirosis were sent to the Pathology Group at the INS for histopathological examination [32]. The tissues were fixed in 10% neutral-buffered formalin for 48 hours. Standard histopathological procedures were followed, including hematoxylin and eosin staining, to assess the quality of tissue preservation. The histological slides, stained with hematoxylin and eosin, were thoroughly examined to identify morphological alterations in various organs. These included liver tissue, where necrosis, steatosis, hemorrhage, Kupffer cell hyperplasia, and inflammatory infiltrate were observed; spleen, showing white pulp hyperplasia, lymphoplasmacytic infiltrate, and vascular congestion; heart, displaying pericardial hemorrhage and inflammatory infiltrate; kidney, indicating acute tubular necrosis, thrombotic microangiopathy, and interstitial nephritis; lung, revealing edema, hemorrhage, and diffuse alveolar damage; and brain, showing edema and cortical hypoxic change. Tissues exhibiting autolytic processes or inadequate samples were excluded from the analysis (n = 3).

### Immunohistochemistry (IHC)

All the tissue samples were tested by IHC for *Leptospira* spp and *Rickettsia* spp., using the CDC protocol [40]. The pathology diagnosis was complemented with serological methods and clinical analysis of the case for classification in the surveillance system. Three to 4-micron thick tissue sections in paraffin-embedded tissue blocks were spread onto Polysine coated sheets. Subsequently, the paraffin was removed with xylol and rehydrated with an ethanolic gradient and subjected to enzymatic digestion with trypsin (0.8 mg/ml in TBS–Tris Buffered saline, Tween 20 0.05%, pH 8.0) for 30 minutes at 37°C, and incubation with 0.9% aqueous hydrogen peroxide for blocking peroxidase and endogenous alkaline phosphatase or 20% aqueous glacial acetic acid for two minutes at 4 °C for blocking. Antigenic recovery was carried out in an aqueous solution (10x EDTA pH 8 in 1:20 dilution) for 30 minutes. After blocking with normal horse serum at 1:20 dilution in TBS for 30 minutes, the tissue was incubated with primary antibody 1:800 in a humid chamber overnight at 4°C (Anti-DENV VS0090, immune mouse ascitic fluid—CDC, Atlanta GA). Tissues were incubated with secondary antibody at a 1:300 dilution in TBS for 20 minutes (anti-mouse biotinylated IgG) and with streptavidin conjugated to alkaline phosphatase (Vector Laboratories) for 30 minutes or using the ABC Kit (Vectastain ABC Kit, Vector laboratories). Development kits for phosphatase (Dako, Liquid Permanent Red) and diaminobenzidine (Zymed) were used. Finally, contrast stains were made with Harris Hematoxylin. Positive and negative controls were included in each staining and were included in each IHC.

### Data analysis

We obtained demographic data from the leptospirosis notification form [32], and medical records were remitted simultaneously with the tissue samples. We used descriptive statistics to show the frequencies of clinical, demographic, and laboratory data from suspected leptospirosis patients. A clinician specialized in infectious diseases, and a group of epidemiologists analyzed all cases to classify diagnoses based on available clinical information, medical records, and laboratory analysis. All data analyses were conducted using EPI-INFO (Version 7.2, CDC, USA) [41]. A map with the distribution of the cases by political division was created using QGIS v3.32.3. We used DIVA-GIS (https://www.diva-gis.org/gdata) to extract boundaries.

## Results

Fresh tissue samples from ninety-two suspected leptospirosis death patients were sent to the laboratory for confirmation by real time-PCR from 2016 to 2019. All cases reported hospitalization, with a mean of 4.4 days length of hospital stay (range 1–28 days). Most of the cases were reported in men (65/92). The mean age was 44 years (range between 10 months and 85 years). Suspected leptospirosis cases were reported in urban (62/92) and urban (30/92) regions (Table 1).

**Table 1:** Characteristics of suspected and confirmed leptospirosis cases in Colombia, 2016–2019.

Characteristics of the cases	Total n = 92	Suspected cases n = 67	Confirmed cases n = 25
	n (%)	n (%)	n (%)
***Year***			
2016	25 (27.1)	16 (23.9)	9 (36.0)
2017	22 (23.9)	16 (23.9)	6 (24.0)
2018	19 (20.7)	16 (23.9)	3 (12.0)
2019	26 (28.3)	19 (28.4)	7 (28.0)
***Sex***			
Women	27 (29.3)	20 (29.9)	7 (28.0)
Men	65 (70.7)	47 (70.1)	18 (72.0)
***Location***			
Rural	30 (32.6)	22 (32.8)	8 (32.0)
Urban	62 (67.4)	45 (67.2)	17 (68.0)
***Age Group***			
1 to 9	3 (3.3)	2 (3.0)	1 (4.0)
10 to 19	11 (12.0)	9 (13.4)	2 (8.0)
20 to 29	14 (15.2)	10 (14.9)	4 (16.0)
30 to 39	14 (15.2)	12 (17.9)	2 (8.0)
40 to 49	12 (13.0)	9 (13.4)	3 (12.0)
50 to 59	14 (15.2)	10 (14.9)	4 (16.0)
60 to 69	10 (10.9)	6 (9.0)	4 (16.0)
≥ 70	14 (15,2)	9 (13.4)	5 (20.0)
***Animal at home***			
Yes	34 (37.0)	22 (32.8)	12 (48.0)
No	8 (8.7)	7 (10.4)	1 (4.0)
No data	50 (54.3)	38 (56.7)	12 (48.0)
***Occupational contact with risk***			
Yes	31 (33.7)	21 (31.3)	10 (40.0)
No	39 (42.4)	28 (41.8)	11 (44.0)
No data	22 (23.9)	18 (26.9)	4 (16.0)
***Sing and symptoms***			
Fever	66 (71.8)	46 (68.7)	20 (80.0)
Jaundice	58 (63.0)	38 (56.7)	20 (80.0)
Myalgia	46 (50.0)	31 (46.3)	15 (60.0)
Headache	33 (35.9)	24 (35.8)	9 (36.0)
Hepatomegaly	30 (32.6)	21 (31.3)	9 (36.0)
Abdominal pain	27 (29.3)	18 (26.9)	9 (36.0)
Dyspnea	23 (25.0)	17 (25.4)	6 (24.0)
Vomit	23 (25.0)	18 (26.9)	5 (20.0)
Arthralgia	18 (19.6)	11 (16.4)	7 (28.0)
Diarrhea	18 (19.6)	11 (16.4)	7 (28.0)
Hypotension	18 (19.6)	10 (14.9)	8 (32.0)
Impaired neurological	18 (19.6)	14 (20.9)	4 (16.0)
Dehydration	13 (14.1)	9 (13.4)	4 (16.0)
Tachycardia	11 (12.0)	5 (7.5)	6 (24.0)
Cough	7 (7.6)	3 (4.5)	4 (16.0)
Hemorrhages	5 (5.4)	2 (3.0)	3 (12.0)
Reticular Pain	4 (4.3)	1 (1.5)	3 (12.0)
Chills	4 (4.3)	2 (3.0)	2 (8.0)
Conjunctivitis	3 (3.3)	1 (1.5)	2 (8.0)
Rash	5 (5.4)	3 (4.5)	2 (8.0)

^a^ Occupational activity with risk of exposure to the *Leptospira* spp. included farmers (n = 20), laborer (n = 10), military (n = 6), mine workers (n = 1), sewage workers (n = 1), and homeless (n = 1).

An etiological agent was detected in 50 (54.3%) of the cases. The distribution of the identified etiologies, as determined by the tests conducted in this study, is presented in Table 2. Leptospirosis was confirmed in 25 (27%) of the fatal cases. These cases tested positive by IHC (n = 15), real-time PCR (n = 14), and MAT (n = 3). Concurrent detection of *Leptospira* spp. was observed across the different methodologies employed. IHC and real-time PCR showed concurrent positive results in six cases, while one case tested positive for both real-time PCR and MAT. In the MAT, agglutination was observed to serogroups Canicola (titer 1:1600), Cynopteri (1:800), and Australis (1:3200) with Copenagheni (1:3200). *Leptospira* spp. was detected as unique pathogen to 14 (15.2%) cases and in 11 cases (12%) *Leptospira* spp. was detected with another etiologic agent (Table 3). Confirmed fatal leptospirosis cases occurred mainly in males (72%), and the fatal cases were reported in all groups of age. The confirmed cases mainly occurred in urban areas (68%). The most frequent signs or symptoms of diagnosed leptospirosis cases were fever (80%), jaundice (80%), myalgia (60%), and headache (36%) (Table 1).

**Table 2:** Etiology detected in the suspected leptospirosis cases.

Etiologic agents	n (%)
Undifferentiated^a^	42 (45.7)
*Leptospira* spp.^b^	14 (15.2)
*Brucella* spp.	8 (8.7)
*Rickettsia* spp.	5 (5.4)
Dengue	4 (4.3)
*Leptospira* spp., and Zika	4 (4.3)
Hepatitis^c^	3 (3.3)
*Leptospira* spp., and *Rickettsia* spp.	3 (3.3)
*Brucella* spp., and *Rickettsia* spp.	1 (1.1)
Dengue and *Rickettsia* spp.	1 (1.1)
*Leptospira* spp., and Dengue	1 (1.1)
*Leptospira* spp., Zika, and Dengue	1 (1.1)
*Leptospira* spp., and *Rickettsia* spp	1 (1.1)
Yellow fever, *Brucella* spp., and Zika	1 (1.1)
Yellow fever and *Rickettsia* spp.	1 (1.1)
Zika	1 (1.1)
*Leptospira* spp., *Rickettsia* spp., and Zika	1 (1.1)
Total	92 (100)

^a^ Undifferentiated was defined as cases in this study that did not show any detection of *Leptospira* spp., or the other etiologies tested by Real-time PCR or pathological examination.

^b^ The detection of *Leptospira* spp. was detected in 25 (27%) cases. We found only *Leptospira spp*. in 14 (15.2%) cases, in 11 (12%) cases *Leptospira* spp., was detected with other etiologies.

^c^ Viral subtype (B-C delta).

**Table 3:** Characteristic of confirmed patients with the detection of more than one pathogen.

Etiology	Case Characteristics					
	Occupation	Sex	Age	Location	Symptoms (days)	Symptoms
Leptospirosis *Rickettsia spp*.	No data	Male	18	Risaralda	2	Jaundice, abdominal pain
	Farmer	Male	75	Risaralda	No data	Myalgia, hepatomegaly, jaundice, abdominal pain.
Leptospirosis *Rickettsia ricketssii*	No data	Male	No data	Antioquia	No data	No data
	Farmer	Male	40	Boyacá	8	Fever, myalgia, headache, Hepatomegaly, jaundice,
Leptospirosis Dengue	Unemployed	Male	54	Tolima	5	Fever, jaundice, Myalgia, headache, hepatomegaly, abdominal pain, chills, dyspnea.
Leptospirosis *Rickettsia spp*.—Zika	Housewife	Female	43	Tolima	7	Malaise, fever, jaundice, diarrhea, vomiting, abdominal pain, and chills.
Leptospirosis- Zika	Housewife	Female	48	Caldas	7	Headache, hepatomegaly, jaundice.
	Military	Male	29	Arauca	7	Fever, myalgia, headache, diarrhea, abdominal pain, dyspnea, cough.
	Farmer	Male	23	Tolima	No data	Fever, myalgia, jaundice, headache, hepatomegaly
Leptospirosis-Zika-Dengue	Waiter	Male	21	Bolivar	5	Fever, rash, Headache, hepatomegaly, abdominal pain, dyspnea, cough.
Dengue-*Rickettsia* spp.	No data	Female	42	Atlántico	7	Myalgia, hepatomegaly, jaundice, abdominal pain, headache, dyspnea.

In 42 (45.7%) cases no infectious agent was detected, most of the cases were reported in males (73.8%, 31/42) and from urban areas (66.6%, 28/42). Within these cases the discharge diagnoses included sepsis (31%, 13/42), unspecified fever (19.4%, 8/42), multiple organ failure (11.9%, 5/42), septic shock and multiple failure (11.9%, 5/42), septic shock (4.8%, 2/42), heart failure (4.8%, 2/42), severe respiratory symptoms (4.8%, 2/42), sepsis and multiple organ failure (4.8%, 2/42), possible poisoning (2.4%, 1/42), leptospirosis (2.4%, 1/42), and liver cancer (2.4%, 1/42).

Among the cases examined, a distinct etiology form leptospirosis was identified in 27% (25/92) cases. *Rickettsia* spp. was detected in thirteen (14.1%, 13/92) fatal cases. Among the tissue samples positive for the *Rickettsia* spp., four samples were positive for *R*. *rickettsii*. We also found *Rickettsia* spp. by PCR in one patient and yellow fever infection by pathology diagnosis in another (S3 Table). *Brucella* spp. was detected in 10 cases (10.9%, 10/92). Also, we detected Zika (8.7%, 8/92), dengue (7.6%, 7/92), hepatitis (3.2%, 3/92), and yellow fever (2.2%, 2/92). None of the cases tested positive for chikungunya or malaria. The characteristics of the cases with an etiology different from leptospirosis is presented in the S3 Table.

**Fig 2:**
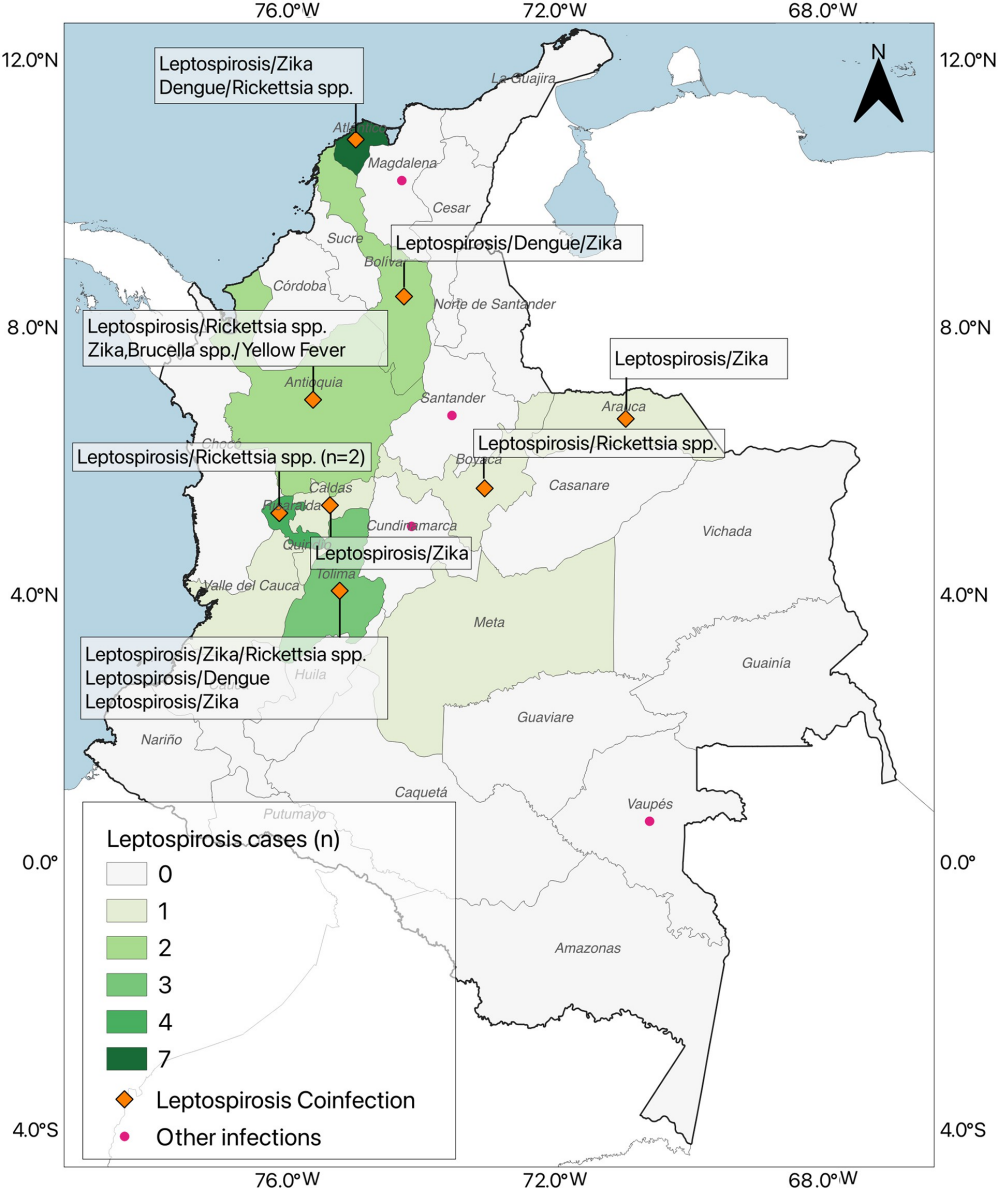
Distribution of the suspected leptospirosis cases in Colombia, 2016–2019. The map was made using QGIS v3.32.3 [QGIS Development Team (2023). QGIS Geographic Information System. https://qgis.org/es/site/forusers/download.html. Map data was obtained from https://www.diva-gis.org/gdata].

The suspected leptospirosis cases were reported from 22 of the 32 political divisions of Colombia. The regions with more suspected leptospirosis cases were Atlántico (18.5%), Sucre (8.7%), Antioquia (7.6%), Tolima (7.6%), Bolivar (7.6%), Risaralda (6.5%), and other (43.5%) (Fig 2). The confirmed leptospirosis cases were reported mainly from Atlántico (28%, 7/25), Risaralda (16%, 4/25), Tolima (12%, 3/25), Antioquia (8%, 2/25), Bolivar (8%, 2/25), and only one confirm cases was reported to Arauca, Boyaca, Caldas, Meta, Quindio, Sucre, and Valle del Cauca.

## Discussion

This study provides data from fatal leptospirosis cases and leptospirosis surveillance in Colombia. Ninety-two suspected fatal leptospirosis cases were reported through laboratory surveillance, of which 27% were confirmed as leptospirosis. Samples were analyzed for other etiologies, obtaining pathological confirmation of dengue (6.9%), hepatitis (3.5%), yellow fever (2.2%), and the molecular detection of *Rickettsia* spp., (14.1%), *Brucella* spp., (10.9%) and Zika (8.7%) from fresh tissue samples. However, we observed a high occurrence of fatal acute undifferentiated febrile illness among the reported cases.

Colombia is an endemic country for leptospirosis. The first leptospirosis case in the country was described in 1957 [42]. Since then, human leptospirosis has been reported in different studies and areas of the country [42]. However, most reported leptospirosis cases are classified as suspected, underscoring the need to obtain laboratory results for a confirmatory diagnosis [31].

Our laboratory confirmation of reported suspected leptospirosis fatal cases shows that even though leptospirosis is endemic in Colombia, the differentiation from other febrile illnesses is still a major challenge, predominantly due to the non-specific febrile syndrome. Early identification of patients with leptospirosis who are at risk for severe disease could help prevent medical complications and death. However, such clinical indicators in leptospirosis patients have not been well elucidated [43]. Many AUFI are preventable, not only leptospirosis. This lack of specificity in the clinical signs and awareness among clinicians could be associated with high morbidity and mortality in Latin American countries [4], resulting in several viral and bacterial etiologies being neglected, misrecognized, and substantially underreported [43–48]. Estimates suggest that approximately half of the AUFI cases remain undiagnosed [23,49].

Although AUFI symptoms may appear similar, conducting thorough patient interviews and critically reviewing medical records are crucial for identifying specific signs that can aid in clinical diagnosis and early treatment [20]. However, it is important to acknowledge that although jaundice may indicate a potential diagnosis of Weil’s syndrome, it can also be observed in other etiologies, including brucellosis, rickettsiosis, and dengue infections. Thus, differentiating the specific cause of jaundice in cases of acute undifferentiated febrile illness poses a significant challenge.

Worldwide, leptospirosis cause approximately 2.90 million Disability Adjusted Life Years (DALYs) (95% CI 1.25–4.54 million) [14,15]. Leptospirosis imposes the largest burden on the most economically productive population. Young adults aged 20–49 have an estimated burden of 1.5 million DALYs (95% CI 0.65–2.32 million), about 52% of the total burden of leptospirosis [15]. Consistent with this evidence, most suspected and confirmed reported leptospirosis patients in our study were in a similar age range, from 20 to 60 years. Due to missing data in the leptospirosis surveillance form submitted along with the tissue samples, we could not associate patients’ occupations with infection. Occupation still matters for leptospirosis infection, particularly in low- and middle-income countries, although the relative importance of occupational risks can be substantially reduced with protective measures [12]. Missing data in surveillance reports hinder infection prevention efforts. Public health officials cannot determine whether exposures are related to occupation, such as livestock farming, or living conditions, such as immersion in water. It is imperative to underscore the importance of epidemiologic information when a leptospirosis case is suspected.

In our study, ten cases tested positive for *Brucella* spp infection. Human brucellosis is characterized as a multi-systemic disease, with the potential to affect any organ or system [7]. Although the mortality rate is low, the disease can lead to significant debilitation and disability, impacting the overall well-being and quality of life of affected individuals [50]. Incorrect diagnosis of the disease can result in clinical complications, which can vary depending on the stage of the disease and the organs affected [48]. Medical complications have been from 20% to 40% [51]. Endocarditis and neuro-brucellosis are the most frequent severe outcomes [50,52]. Multifocal presence of abscesses or nodules in the liver, lung, and pleura [51], spontaneous bacterial peritonitis in apparently healthy patients [52], acute liver failure [53], fatal cardiac arrest [54], and multi-organ failure [55] have also been described as fatal complications due to brucellosis. Low- and middle-income countries have a significant burden of brucellosis [56,57], with a high incidence of human brucellosis in South America [57**–**59]. However, in Colombia, it is important to note that epidemiological surveillance for brucellosis remains limited, the focus of brucellosis surveillance is primarily on livestock. This is due to the implementation of a control, prevention, and eradication program for brucellosis in animals by the Colombian Agriculture Institute since 2002. As a result, surveillance efforts directed towards human brucellosis are relatively restricted, which may contribute to underestimating the true burden of the disease in the human population [60]. The detection of *Brucella* spp. in fatal cases with suspected leptospirosis underscores the significance of adopting an integrated approach to disease surveillance that encompasses both human health and veterinary services. This integrated approach allows for a comprehensive understanding of zoonotic diseases like brucellosis and leptospirosis, facilitating early detection, prevention, and appropriate management strategies.

By collaborating and sharing information between human health and veterinary sectors, we can effectively address the challenges posed by these interconnected diseases and enhance public health outcomes [61]. The relatively high proportion of brucellosis found in our study could be associated with the consumption of unpasteurized dairy products in many Colombia departments [60]. A complementary explanation may be clinical misrecognition, as found elsewhere [54], which could be explained by a lack of awareness from limited surveillance [48]. It is crucial to strengthen awareness of brucellosis, particularly by including it in the differential diagnosis of febrile illnesses among clinicians in Colombia. This approach is essential for preventing misrecognition and underreporting of human brucellosis cases. Given that Colombia is an endemic region with favorable conditions for the spread of this zoonotic disease, increasing clinician awareness can lead to timely diagnosis, appropriate treatment, and improved surveillance and reporting of brucellosis cases. Ultimately, this proactive approach will contribute to better control and prevention of brucellosis in the country.

Remarkably, out of ten brucellosis cases, two were identified as coinfections with other etiologies (brucellosis-Zika and brucellosis-rickettsiosis). Previous cases of brucellosis-dengue coinfection have been reported [62]. These coinfections can result in substantial liver damage, increasing the risk of death. Leptospirosis may also be missed by erroneous clinical or laboratory diagnosis of arboviruses, and coinfections can increase the likelihood of clinical complications, mismanagement, and death [63**–**66]. In Colombia, previous studies have evidenced the diagnostic challenge of leptospirosis and rickettsiosis in febrile syndrome in endemic areas [67,68]. In a retrospective study, elevated antibodies to *Leptospira* spp. were detected in deceased patients who had a confirmed diagnosis of dengue, indicating a potential coinfection. Additionally, some patients initially suspected to have dengue were later found to be positive for leptospirosis, suggesting that the presence of dengue may have masked the diagnosis of leptospirosis [69]. Cardona-Ospina *et al*., presented cases indicating that concurrent viral co-infections with leptospirosis might have contributed to fatal outcomes [68]. Similarly, Ramírez-García R *et al* [67] reported a case of concurrent leptospirosis and rickettsiosis infection in a patient with a high-risk occupational history in an endemic area for tropical diseases. The patient exhibited progressive and ultimately fatal clinical symptoms [67]. Consequently, leptospirosis should be considered as a potential cause of the febrile syndrome, not only as an isolated agent but also as a possible co-infection that can have serious implications for patient health and may be associated with increased mortality rates [30,65,67,69].

Since the first human rickettsiosis infection was reported in Colombia in 1934, there have been several outbreaks with a case fatality rate reaching up to 54% in endemic areas [70,71]. Nevertheless, the disease burden of rickettsiosis is still unknown. In 2012, two fatal cases of human rickettsiosis in young adults were reported in Cundinamarca, Colombia. The cause of death was evidenced by the presence of *R*. *rickettsii*. in the microvascular endothelium of various organs, including the liver, spleen, lungs, and brain [72]. More recently, Quintero-Velez et al. [73] reported another fatal case of *R*. *rickettsii* infection in a child where both conventional PCR and histopathological studies confirmed the pathogen’s presence in *post-mortem* samples from various organs. Human rickettsiosis is not included in the differential diagnoses of febrile syndromes in Colombia and does not require reporting. We found positive tissue samples for *Rickettsia* spp. by real-time PCR in thirteen cases of our sample, which suggests rickettsiosis may be another important febrile illness in Colombia. Several species of *Rickettsia* have been found in Colombia [70,71,73,74]. Only four of 13 patients with *Rickettsia* spp. were identified as *R*. *rickettsii*. The identification of species for all cases should be attempted in future studies. Because of incomplete clinical reports and case information, we cannot conclude that rickettsiosis was the cause of death in those patients. We also observed *Rickettsia* spp. in confirmed leptospirosis cases; this coinfection has been associated with severe leptospirosis [75].

Our study has four main limitations. First, data collection was made by retrospective passive surveillance through the national surveillance of fatal leptospirosis reports. We could not recollect all information from patients as surveillance forms sent with the tissue samples were often incomplete. Due to missing data, we could not establish a correlation between confirmed cases and epidemiological risk factors such as occupational or contact activities with contaminated sources. Second, laboratory surveillance also had limitations related to the shipment of serum samples. Confirmed leptospirosis cases were based on fresh tissue samples for PCR and formalin-fixed tissues using the IHC. Since serum samples were not available, we could not use a microscopic agglutination test to identify seroconversion and identify *Leptospira* spp. serogroups. However, detection by PCR has been used as a tool for the confirmation of acute and fatal leptospirosis diagnosis, as the pathogenesis is related to the presence of bacteria in tissues. PCR can support the assessment of the incidence and epidemiology of leptospirosis where serology studies are unavailable [76–79]. In future studies, it is essential to incorporate phylogenetic studies and the sequencing of positive results, particularly through molecular diagnostic tests. These complementary measures will provide a comprehensive understanding of infectious entities associated with fatal cases. Third, we could not establish that pathogens other than leptospirosis detected in our samples were the primary cause of death because of missing clinical and serological data. However, our results highlight the circulation of pathogens that are not considered a notifiable disease in current surveillance. This finding serves as a valuable contribution to raising clinical awareness regarding pathologies like rickettsiosis and brucellosis, alongside more common causes of AUFI in Colombia. It highlights the importance of considering these diseases in the differential diagnosis when evaluating patients presenting with AUFI symptoms. However, it is important to note that our study did not include testing for other significant infections that are prevalent in tropical areas of Latin America as the Hantavirus, Mayaro, Oropouche, and West Nile virus [4]. Further research and surveillance efforts are needed to address the broader spectrum of infections in these regions and enhance our understanding of their epidemiology and clinical management.

In conclusion, we found that leptospirosis was confirmed in 27% of 92 *postmortem* AUFI cases reported as suspected leptospirosis cases through the national leptospirosis surveillance in Colombia. Undifferentiated febrile illness was reported to 45.7%, underscoring diagnostic limitations common in the tropics, and the need to recognize local pathogens and clinical characteristics to perform early presumptive diagnosis in patients with acute undifferentiated febrile illness. Our results underscore the relatively high presence of clinically unsuspected infectious diseases such as brucellosis and rickettsiosis. We hope our results will help raise awareness among medical practitioners and public health officials of diagnostic difficulties, possibilities, and differential diagnoses of acute hemorrhagic fevers and AUFI cases. We found a substantial proportion of coinfections with other pathogens, which may lead to clinical complications and deaths. Our results highlight some of the challenges to accurately identify AUFI cases due to similar clinical presentation and mimicking of signs and symptoms with other febrile illnesses. Delayed diagnosis or inappropriate medical management may lead to preventable medical complications or death among leptospirosis patients. Improved surveillance and diagnosis are crucial to provide early treatment, avoid complications, and reduce preventable deaths. Also, raising awareness about human leptospirosis in the medical community will allow early and timely diagnosis to achieve early treatment and reduce mortality and associated complications. The unfortunate lack of understanding regarding the elevated risks of mortality posed by these ailments is disheartening, as illustrated by their retrospective identification within our locality. This unsettling reality serves as a poignant reminder that fatal fevers persist without timely antemortem diagnoses.

## Supporting information

S1 Table Serogroups included in the panel for the MAT test, which is used for case confirmation and surveillance of circulating serovars in the country by the national reference laboratory. (DOCX)

S2 Table PCR assay reactants, genetic blanks, and controls. (DOCX)

S3 Table Demographics and characteristics of the identified non-leptospirosis cases. (DOC)
