# Supplementary material for: Fatal acute undifferentiated febrile illness among clinically suspected leptospirosis cases in Colombia, 2016–2019
Source: PLoS Negl Trop Dis. 2023 Oct 16;17(10):e0011683. doi: 10.1371/journal.pntd.0011683 (PMC10602388; doi:10.1371/journal.pntd.0011683)
Supplement: S1 Table — (DOCX) [file pntd.0011683.s001.docx]

**S1 Table.** Serogroups included in the panel for the MAT test, which is used for case confirmation and surveillance of circulating serovars in the country by the national reference laboratory.

| **N°** | **Specie** | **Serogroup** | **Serovar** | **Strain ^a^** |
| --- | --- | --- | --- | --- |
| 1 | *L. interrogans* | Australis | Australis | Ballico |
| 2 | *L. interrogans* | Australis | Bratislava | Jez Bratislava |
| 3 | *L. interrogans* | Autumnalis | Autumnalis | Akyyami a |
| 4 | *L. interrogans* | Autumnalis | Rachmati | Rachmat |
| 5 | *L. interrogans* | Bataviae | Bataviae | Swart |
| 6 | *L. interrogans* | Icterohaemorrhagiae | Birkini | Birkini |
| 7 | *L. interrogans* | Canicola | Canicola | Hond Ultrecht IV |
| 8 | *L. interrogans* | Hebdomadis | Hebdomadis | Hebdomadis |
| 9 | *L. interrogans* | Icterohaemorrhagiae | Copenhageni | M20 |
| 10 | *L. interrogans* | Icterohaemorrhagiae | Icterohaemorrhagiae | RGA |
| 11 | *L. interrogans* | Pomona | Pomona | Pomona |
| 12 | *L. interrogans* | Pomona | Proechymis | 1161U |
| 13 | *L. interrogans* | Pyrogenes | Pyrogenes | Salinem |
| 14 | *L. interrogans* | Sejroe | Wolffi | 3705 |
| 15 | *L. interrogans* | Sejroe | Hardjo | Hardjoprajitno |
| 16 | *L. interrogans* | Sejroe | Saxkoebing | Mus 24 |
| 17 | *L. borgpetersenii* | Ballum | Ballum | Mus 127 |
| 18 | *L. borgpetersenii* | Ballum | Castellonis | Castellon 3 |
| 19 | *L. borgpetersenii* | Javanica | Javanica | Veldrat Batavia 46 |
| 20 | *L. borgpetersenii* | Javanica | Poi | Poi |
| 21 | *L. borgpetersenii* | Sejroe | Sejroe | M 84 |
| 22 | *L. borgpetersenii* | Tarassovi | Tarassovi | Perepelitsin |
| 23 | *L. weilii* | Celledoni | Celledoni | Celledoni |
| 24 | *L. noguchii* | Panama | Panama | CZ214 |
| 25 | *L.kirschneri* | Cynopteri | Cynopteri | 3522C |
| 26 | *L.kirschneri* | Grippotyphosa | Grippotyphosa | Moskva V |
| 27 | *L. santarosai* | Sejroe | Gorgas | 1413U |
| 28 | *L. santarosai* | Sejroe | Guaricura | Bov G |
| 29 | *L. santarosai* | Shermani | Shermani | 1342K |
| 30 | *L. biflexa* | Semaranga | Patoc | Patoc I |

**^a^** Strains were obtained through commercial purchase from the Royal Tropical Institute Center, Amsterdam.
