# Supplementary material for: Fatal acute undifferentiated febrile illness among clinically suspected leptospirosis cases in Colombia, 2016–2019
Source: PLoS Negl Trop Dis. 2023 Oct 16;17(10):e0011683. doi: 10.1371/journal.pntd.0011683 (PMC10602388; doi:10.1371/journal.pntd.0011683)
Supplement: S2 Table — (DOCX) [file pntd.0011683.s002.docx]

**S2 Table**. PCR assay reactants, genetic blanks, and controls.

| **Agent** | **Ref** | **Target** | **Internal control** | **Positive control** |
| --- | --- | --- | --- | --- |
| *Leptospira spp.* | 33 | lipL32 | TaqMan™ Exogenous Internal Positive Control Reagents, Applied Biosystems | Leptospira interrogans, serovar Icterohaemorrhagiae, strain RGA (10pg/ul) |
| *Rickettsia spp.* | 34 | 23S rRNA | Ribonucleasa (RNasa) | DNA kindly provided by the doctor Salim Mattar from the University of Cordoba, Colombia, South America |
| *Rickettsia rickettsii* | 34 | Encoding hypothetical protein A1G_04230 |  |  |
| *Brucella spp.* | 35 | IS711 insertion sequence |  | Strain A19 |
| *Dengue* | 36 | CDC Trioplez Real-time RT-PCR Assay (Trioplex rRt-PCR) | Human endogenous ribonuclease P (RP) | CDC Trioplex rRt_PCR Positive Control Set-Packge Insert KT0167 |
| *Chikungunya* | 36 |  |  |  |
| *Zika* | 36 |  |  |  |
| *Plasmodium spp*. | 37, 38 | SSrRNA | β-globin Gene was used as an Internal Control for DNA Extraction | *P. falciparum* Colombian strain.  *P. vivax* Colombian strain. |
| *P. falciparum* | 39 | rPLU3 and rPLU4 |  |  |
| *P. vivax* |  |  |  |  |

Note: No Template Control (NTC) was used to control the contamination or improper function of assay reagents in the cases of resulting in false positive results. NTC reactions included PCR-grade water instead of the specimen in DNA and RNA for each reaction mixture.
