# Supplementary material for: Fatal acute undifferentiated febrile illness among clinically suspected leptospirosis cases in Colombia, 2016–2019
Source: PLoS Negl Trop Dis. 2023 Oct 16;17(10):e0011683. doi: 10.1371/journal.pntd.0011683 (PMC10602388; doi:10.1371/journal.pntd.0011683)
Supplement: S3 Table — (DOC) [file pntd.0011683.s003.doc]

**S3 Table.** Demographics and characteristics of the identified non-leptospirosis cases.

| **Cases** | **Year** | **Location** | **Area of ocurrence** | **Gender** | **Age (Year)** | **Animal at home** | **Symptoms (days)** | **Hospitalization (days)** | **Symptoms** | **Coinfection** | **Clinician**  **discharge diagnoses** | |
| --- | --- | --- | --- | --- | --- | --- | --- | --- | --- | --- | --- | --- |
| ***Rickettsia spp. (n=13)*** | | | | | | | | | | | |  |
| 1 | 2016 | Antioquia | Urban | Male | 3 | ND | 2 | 4 | ND | *Leptospira* spp- Rickettsia spp | Sepsis | |
| 2 | 2016 | Vaupés | Urban | Male | 18 | Yes | 15 | 1 | Fever, myalgia, headache, jaundice | Yellow fever-*Rickettsia* spp | Sepsis | |
| 3 | 2016 | Boyacá | Rural | Male | 40 | Yes | 8 | 1 | Fever, myalgia, headache, jaundice, hepatomegaly | *Leptospira* spp | Unspecified fever | |
| 4 | 2016 | Tolima | Urban | Female | 37 | Yes | 3 | 1 | Fever, myalgia, headache, hepatomegaly |  | Sepsis | |
| 5 | 2017 | Atlántico | Urban | Female | 42 | Yes | 7 | 7 | Headache, hepatomegaly, jaundice | Dengue | Dengue | |
| 6 | 2017 | Sucre | Urban | Female | 42 | No | 28 | 28 | Fever, myalgia, headache | *Brucella* spp | Septic Shock- Multiple Organ Failure | |
| 7 | 2017 | Atlántico | Urban | Male | 40 | ND | 1 | 6 | ND |  | Possible hematologic neoplasm | |
| 8 | 2017 | Risaralda | Rural | Male | 18 | ND | 2 | 1 | Jaundice | *Leptospira* spp | Unspecified fever | |
| 9 | 2018 | Magdalena | Rural | Male | 38 | ND | 7 | 1 | Fever, myalgia, jaundice, rash |  | Sepsis | |
| 10 | 2018 | Quindio | Urban | Female | 81 | No | 9 | 5 | Fever, myalgia, headache, jaundice |  | Unspecified fever | |
| 11 | 2018 | Risaralda | Urban | Male | 75 | ND | 4 | 4 | Hepatomegaly, jaundice |  | Unspecified fever | |
| 12 | 2019 | Tolima | Urban | Female | 43 | ND | 7 | 5 | Fever, myalgia, headache, jaundice | Zika - *Leptospira* spp - Rickettsia spp | Septic Shock - Multiple Organ Failure | |
| 13 | 2019 | Boyacá | Rural | Male | 27 | Yes | 6 | 6 | Fever, myalgia, headache, rash |  | Meningitis | |
| ***Brucella spp. (n=10)*** | | | | | | | | | | | | |
| 1 | 2016 | Antioquia | Urban | Male | 3 | ND | 2 | 4 | ND | *Leptospira* spp- Rickettsia spp | Sepsis | |
| 1 | 2016 | Arauca | Rural | Male | 35 | No | 8 | 1 | Asthenia |  | Unspecified fever | |
| 2 | 2016 | Atlántico | Urban | Male | 71 | Yes | 19 | 15 | Jaundice,  Myalgia, asthenia |  | Unspecified fever | |
| 3 | 2016 | Antioquia | Urban | Male | 25 | ND | 7 | 6 | Fever, jaundice, Myalgia, headache | Zika and Yellow Fever | Unspecified fever | |
| 4 | 2016 | Atlántico | Urban | Male | 51 | Yes | 15 | 5 | Jaundice, myalgia, headache |  | Unspecified fever | |
| 5 | 2017 | Sucre | Urban | Female | 42 | No | 28 | 28 | Fever, myalgia headache | *Brucella* spp., and *Rickettsia* spp., | Septic Shock - Multiple Organ Failure | |
| 6 | 2018 | Cundinamarca | Urban | Female | 42 | ND | 39 | 6 | Fever, jaundice Asthenia, Headache |  | Multiple Organ Failure | |
| 7 | 2018 | Santander | Urban | Male | 85 | ND | 29 | 12 | Fever |  | Multiple Organ Failure | |
| 8 | 2018 | Sucre | Urban | Female | 19 | ND | 10 | 1 | Fever, jaundice, asthenia |  | Liver failure | |
| 9 | 2019 | Bolívar | Urban | Male | 67 | ND | 10 | 6 | Fever, jaundice, mialgia, asthenia |  | Unspecified fever | |
| 10 | 2019 | Atlántico | Urban | Male | 32 | Yes | 10 | 1 | Fever, jaundice, mialgia, headache |  | Unspecified fever | |
| **Zika (n=8)** | | | | | | | | | | | | |
| 1 | 2016 | Antioquia | Rural | Male | 25 | ND | 7 | 6 | Fever, myalgia, headache, jaundice, vomit | Yellow fever-Brucella spp | Unspecified fever | |
| 2 | 2016 | Caldas | Urban | Female | 48 | Yes | 7 | 7 | Headache, hepatomegaly, jaundice | *Leptospira* spp | Leptospirosis | |
| 3 | 2016 | Arauca | Rural | Male | 29 | ND | 7 | 1 | Fever, myalgia, headache, | *Leptospira* spp | Sepsis | |
| 4 | 2016 | Tolima | Urban | Male | 68 | Yes | 6 | 5 | Fever, myalgia, |  | Unspecified fever | |
| 5 | 2016 | Atlántico | Urban | Female | 75 | ND | 6 | 2 | Fever, myalgia, jaundice | *Leptospira* spp (Canicola 1:1600) | Sepsis | |
| 6 | 2017 | Bolívar | Urban | Male | 21 | ND | 5 | 1 | Fever, hepatomegaly, rash | *Leptospira* spp | Dengue | |
| 7 | 2019 | Tolima | Urban | Female | 43 | ND | 7 | 5 | Fever, jaundice. vomit | *Leptospira* spp- *Rickettsia* spp | Septic Shock - Multiple Organ Failure | |
| 8 | 2019 | Tolima | Urban | Male | 23 | ND | 3 | 1 | Fever, myalgia, jaundice, conjunctivitis | *Leptospira* spp | Septic Shock | |
| **Dengue (n=7)** | | | | | | | | | | | |  |
| 1 | 2017 | Atlántico | Urban | Female | 42 | Yes | 7 | 7 | Myalgia, headache, hepatomegaly, jaundice, dyspnoea | *Rickettsia* spp | Dengue | |
| 2 | 2017 | Bolívar | Urban | Male | 21 | No data | 5 | 1 | Fever, headache, hepatomegaly, rash, abdominal pain, dyspnoea, cough, hemorrhage | *Leptospira spp-Zika* | Dengue | |
| 3 | 2019 | Bolívar | Rural | Male | 1 | No data | 1 | 1 | Fever |  | Pneumonia | |
| 4 | 2019 | Tolima | Rural | Male | 35 | Yes | 8 | 6 | Fever, mialgia, hepatomegaly, jaundice, vomit, abdominal pain, |  | Unspecified fever | |
| 5 | 2019 | Tolima | Urban | Male | 54 | Yes | 5 | 1 | Fever, mialgia, hepatomegaly, jaundice, abdominal pain, dyspnoea | *Leptospira* spp | Septic Shock - Multiple Organ Failure | |
| 6 | 2019 | Sucre | Rural | Female | 19 | No | 14 | 14 | Fever, headache, hepatomegaly, jaundice |  | Unspecified fever | |
| 7 | 2019 | Antioquia | Urban | Male | 33 | Yes | 4 | 1 | Fever, Jaundice, diarrhea, vomit, abdominal pain, dyspnoea |  | Septic Shock | |

ND: No data
